# Human eukaryotic initiation factor 4E (eIF4E) and the nucleotide-bound state of eIF4A regulate eIF4F binding to RNA

**DOI:** 10.1016/j.jbc.2022.102368

**Published:** 2022-08-11

**Authors:** Mario Servulo Izidoro, Masaaki Sokabe, Nancy Villa, William C. Merrick, Christopher S. Fraser

**Affiliations:** 1 Department of Molecular and Cellular Biology, College of Biological Sciences, University of California, Davis, California, USA; 2 Department of Biochemistry, School of Medicine, Case Western Reserve University, Cleveland, Ohio, USA

**Keywords:** eIF4E, eIF4A, eIF4G, eIF4F, translation initiation, cooperativity, RNA, BTE, Barley yellow dwarf virus–like translation element, eIF, eukaryotic initiation factor, FL, fluorescein labeled, MESNA, sodium 2-mercaptoethanesulfonate, m7G, 5′ 7-methyl guanosine, Ni–NTA, nickel–nitrilotriacetic acid, PIC, preinitiation complex

## Abstract

During translation initiation, the underlying mechanism by which the eukaryotic initiation factor (eIF) 4E, eIF4A, and eIF4G components of eIF4F coordinate their binding activities to regulate eIF4F binding to mRNA is poorly defined. Here, we used fluorescence anisotropy to generate thermodynamic and kinetic frameworks for the interaction of uncapped RNA with human eIF4F. We demonstrate that eIF4E binding to an autoinhibitory domain in eIF4G generates a high-affinity binding conformation of the eIF4F complex for RNA. In addition, we show that the nucleotide-bound state of the eIF4A component further regulates uncapped RNA binding by eIF4F, with a four-fold decrease in the equilibrium dissociation constant observed in the presence *versus* the absence of ATP. Monitoring uncapped RNA dissociation in real time reveals that ATP reduces the dissociation rate constant of RNA for eIF4F by ∼4-orders of magnitude. Thus, release of ATP from eIF4A places eIF4F in a dynamic state that has very fast association and dissociation rates from RNA. Monitoring the kinetic framework for eIF4A binding to eIF4G revealed two different rate constants that likely reflect two conformational states of the eIF4F complex. Furthermore, we determined that the eIF4G autoinhibitory domain promotes a more stable, less dynamic, eIF4A-binding state, which is overcome by eIF4E binding. Overall, our data support a model whereby eIF4E binding to eIF4G/4A stabilizes a high-affinity RNA-binding state of eIF4F and enables eIF4A to adopt a more dynamic interaction with eIF4G. This dynamic conformation may contribute to the ability of eIF4F to rapidly bind and release mRNA during scanning.

The eukaryotic initiation factor 4F (eIF4F) complex binds the 5′ 7-methyl guanosine (m^7^G) cap found on all cellular mRNAs and is comprised of the cap-binding protein, eIF4E, the DEAD-box helicase, eIF4A, and the scaffold protein, eIF4G (1, 2). The interaction of the eIF4F complex with m^7^G cap is important in recruiting the 5′ end of an mRNA to the 43S preinitiation complex (PIC). The eIF4F complex functions to also unwind secondary structure in the mRNA 5′ UTR, an activity that is attributed to the eIF4A helicase component (3, 4, 5). Thus, eIF4F possesses both cap-dependent and cap-independent functions that are important during mRNA recruitment to the 48S complex and its subsequent scanning/translocation to select an initiation codon (reviewed in Refs. (1, 6, 7)). The cap-dependent and cap-independent functions of human eIF4F are both regulated by the eIF4E component. For its cap-dependent function, eIF4E specifically binds to the m^7^G cap structure, which initially tethers eIF4F to the 5′ end of an mRNA (8, 9, 10). Regarding its cap-independent function, the binding of eIF4E to an autoinhibitory domain in human eIF4G promotes the helicase activity of eIF4A (11, 12). Interestingly, it has recently been reported that the autoinhibitory domain in eIF4G is not conserved in *Saccharomyces cerevisiae*, at least in its ability to regulate the helicase activity of eIF4A (13). Yet, it is noteworthy to mention that the equilibrium dissociation constant of eIF4A from eIF4G is decreased roughly 10-fold in the presence of eIF4E in *S. cerevisiae*, indicating a strong thermodynamic coupling between eIF4E and eIF4A binding to eIF4G (14). It has also been shown that eIF4A becomes a processive helicase in the presence of eIF4G and eIF4B by a mechanism that is also cap independent (15).

During initiation, the eIF4F complex must coordinate interactions between eIF4E, eIF4G, eIF4A, ATP, and RNA. This coordination is important in forming a thermodynamically stable complex that can promote mRNA recruitment to form the 48S complex. It is also critical that the eIF4F complex can cycle between binding and releasing RNA so that secondary structure can be melted and for scanning/translocation to occur after 48S complex formation. Toward understanding this regulation, studies have shown that ATP binding and hydrolysis produces a cycle of conformational and RNA-affinity changes in eIF4A in the absence of other components (16, 17, 18). Single-molecule assays have provided insight into how the conformational changes in eIF4A are regulated by eIF4G and the eIF4B accessory protein. The binding of eIF4G to eIF4A guides a transition between a half-open conformation of eIF4A and a fully closed conformation (19). The fully closed conformation of eIF4A becomes further stabilized in the presence of both eIF4G and eIF4B (20). The stabilization of the closed conformation and the cycling between closed and half-open conformations is consistent with the finding that eIF4A becomes a processive helicase in the presence of both eIF4G and eIF4B (15).

Most biophysical assays used to study the activity of eIF4A in the presence of human eIF4G have used a minimum HEAT repeat domain of eIF4G that only interacts with eIF4A. While the minimum eIF4G domain can reveal many fundamental aspects by which eIF4G regulates eIF4A activity, it is likely to miss some important aspects of regulation in the eIF4F complex. Consistent with this, our previous work revealed that the binding of eIF4E to an autoinhibitory domain in human eIF4G strongly promotes the helicase activity of eIF4A (11, 12). Interestingly, the binding of eIF4E to the autoinhibitory domain of plant eIF4G increases the affinity of eIF4G for a 3′ cap-independent Barley yellow dwarf virus–like translation element (BTE) by 2.5-fold to 6-fold depending on the eIF4G truncation used (21). This implies that this domain is important in regulating RNA binding to eIF4F, although it should be noted that no eIF4A was present in the study. Since the affinity of plant eIF4G to the BTE has such a high affinity in the absence of eIF4E (40 nM), it is also not clear what the increase in affinity in the presence of eIF4E means for the regulation of RNA binding by plant eIF4G. A single-molecule study using yeast components revealed that eIF4E binds to the m^7^G cap with an increased association rate in the presence of full-length eIF4G, which was appreciably different to the association rate that was observed when using a truncated eIF4G protein (22). Nevertheless, it was recently shown that the m^7^G cap does not change the observed helicase activity of yeast eIF4F (13). Taken together, these different studies indicate that the mechanism by which eIF4F binds and releases mRNA as a complex during the different stages of initiation is still poorly defined.

Here, we have employed steady-state and real-time anisotropy assays using a highly purified reconstituted human system to explore how interactions between eIF4E, eIF4G, eIF4A, and ATP regulate RNA binding and release from eIF4F. We show that the binding of eIF4E to an autoinhibitory domain in eIF4G generates a high-affinity RNA-binding conformation of the eIF4F complex. The interaction between eIF4E and the autoinhibitory domain in eIF4G also promotes a more dynamic eIF4A-binding state, which provides a plausible mechanism to explain how eIF4E promotes the helicase activity of eIF4A. We also demonstrate that ATP binding and hydrolysis generates a cycle of dramatic RNA affinity and lifetime changes, enabling eIF4F to rapidly bind and release RNA. Overall, our data provide fundamental insights into how changes in the rate of eIF4F binding and release from uncapped RNA enable the 48S complex to efficiently scan/translocate on mRNA during initiation.

## Results

### Cap-independent role of eIF4E in RNA binding by the cap-binding complex eIF4F

To investigate the thermodynamic framework for human eIF4F binding to RNA, we prepared different length fluorescent-labeled uncapped RNAs by 3′-end modification with fluorescein-5-thiosemicarbazide, as described in the Experimental procedures section. To determine an appropriate length of RNA that can provide a binding site for human eIF4F, we produced fluorescein labeled (FL) 20-nt, 32-nt, 42-nt, and 54-nt length CAA-repeat containing RNAs, as described in the Experimental procedures section. To establish a fluorescence anisotropy–binding assay, we used a previously characterized truncation of human eIF4G that mimics a poliovirus 2A protease cleaved form of the protein (including amino acids 682–1599; eIF4G_682–1599_) (12). We examined the binding of each RNA to an eIF4G_682–1599_–eIF4A–ATP complex by titrating eIF4G_682–1599_ into a fixed amount of each CAA-FL RNA in the presence of a fixed amount of eIF4A–ATP. The fixed amount of eIF4A–ATP used was based on a previously published binding affinity and as described in the Experimental procedures section. The change in fluorescence anisotropy that was specific to the eIF4G_682–1599_–eIF4A–ATP complex was measured. A strong anisotropy increase upon eIF4G_682–1599_–eIF4A–ATP titration was observed, indicating productive binding to form a complex (Fig. S1 and Table 1). Converting anisotropy values into the fraction of CAA-FL bound at each eIF4G_682–1599_–eIF4A–ATP concentration yields an equilibrium dissociation constant (*K*_*d*_) of 1763, 375, 321, and 260 nM for CAA-20-FL, CAA-32-FL, CAA-42-FL, and CAA-54-FL, respectively (Fig. S1, also see Table 1 for *K*_*d*_ values). We decided to use CAA-42-FL for the rest of our study since it appears to sufficiently provide a complete binding site for an eIF4G_682–1599_–eIF4A–ATP complex.

**Table 1:** Summary of equilibrium-binding parameters

Labeled molecule	Complex bound	Nucleotide	*K*_*d*_^a^ (nM)	*K*_*i*_^b^ (nM)	*r*_free_^c^	*r*_bound_^d^	Δ*r*_max_^e^
CAA42-FL	eIF4G_682–1104_ ± eIF4A	—	3885 ± 124		0.088 ± 0.001	0.194 ± 0.002	0.106 ± 0.001
		ADP	2646 ± 65		0.091 ± 0.001	0.194 ± 0.002	0.103 ± 0.001
		ATP	735 ± 26		0.152 ± 0.001	0.256 ± 0.001	0.103 ± 0.001
	eIF4G_682–1599_ ± eIF4A	—	1056 ± 101	3925 ± 519	0.106 ± 0.001	0.175 ± 0.001	0.069 ± 0.002
		ADP	1676 ± 267		0.105 ± 0.001	0.157 ± 0.002	0.052 ± 0.001
		ATP	321 ± 71	1194 ± 154	0.151 ± 0.001	0.213 ± 0.005	0.062 ± 0.004
	eIF4G_557–1599_ ± eIF4A ± eIF4E	—	520 ± 15		0.108 ± 0.001	0.243 ± 0.001	0.135 ± 0.001
		ADP	1018 ± 22		0.096 ± 0.001	0.231 ± 0.001	0.135 ± 0.001
		ATP	136 ± 11		0.143 ± 0.002	0.242 ± 0.002	0.099 ± 0.002
	eIF4G_557–1599_ ± eIF4A	ATP	∼7000^f^		0.114 ± 0.001	0.242 ± 0.002	0.128 ± 0.001
	eIF4A	ATP	4127 ± 767		0.103 ± 0.002	0.196 ± 0.010	0.092 ± 0.008
		ADP	>>10,000		0.090 ± 0.001	ND	ND
CAA20-FL	eIF4G_682–1599_ ± eIF4A	ATP	1763 ± 322		0.139 ± 0.001	0.165 ± 0.001	0.026 ± 0.001
CAA32-FL	eIF4G_682–1599_ ± eIF4A	ATP	375 ± 22		0.152 ± 0.003	0.206 ± 0.001	0.054 ± 0.003
CAA54-FL	eIF4G_682–1599_ ± eIF4A	ATP	260 ± 15		0.153 ± 0.001	0.195 ± 0.001	0.042 ± 0.001
CAA42–5′FL	eIF4G_682–1599_ ± eIF4A	ATP	273 ± 22		0.193 ± 0.002	0.255 ± 0.001	0.062 ± 0.003
eIF4A-FL	eIF4G_557–1599_	—	53 ± 3		0.135 ± 0.001	0.180 ± 0.002	0.045 ± 0.002
	eIF4G_557–1599_ ± eIF4E	—	102 ± 7		0.139 ± 0.001	0.180 ± 0.002	0.041 ± 0.002

ND, not determined.

a Equilibrium dissociation constants determined by titrating the mRNA-Fl with the binding partners under the experimental conditions.

b Equilibrium dissociation constants determined by titrating the unlabeled RNA to the preformed complex including CAA42-FL under the experimental condition.

c Anisotropy of the mRNA-Fl prior to addition of any binding partner under the experimental conditions.

d Anisotropy of the mRNA-Fl in the bound state.

e Difference between *r*_free_ and *r*_bound_, representing the maximum anisotropy change.

f The value was estimated from a titration ranging from 0 to 2000 nM eIF4G_557–1599_.

To determine the contribution of eIF4G_682–1599_ on the binding of CAA-42-FL to the eIF4G_682–1599_–eIF4A–ATP complex, we determined the equilibrium dissociation constant of CAA-42-FL to eIF4A–ATP. A robust anisotropy increase upon eIF4A–ATP titration was observed, indicating that a CAA-42-FL–eIF4A–ATP complex can be monitored using our anisotropy assay (Fig. S2 and Table 1). The CAA-42-FL binds to eIF4A–ATP with a *K*_*d*_ of 4127 nM, which is roughly an order of magnitude greater than to eIF4G_682–1599_–eIF4A–ATP (321 nM). To determine whether the fluorophore conjugated to CAA-42-FL affects the interaction with eIF4G_682–1599_–eIF4A–ATP, we titrated a preformed eIF4G_682–1599_–eIF4A–ATP–CAA-42-FL complex with increasing concentrations of a nonlabeled CAA-42 RNA (Fig. S3*A*). The inhibition constant of this interaction was found to be 1194 nM, which is roughly 3.7-fold larger than the equilibrium dissociation constant (321 nM; Table 1). This discrepancy may reflect that the fluorophore has a modest effect on RNA binding. However, we feel that this is unlikely to be the case since 5′- and 3′-end labeled RNAs possess the same affinity for an eIF4G_557–1599_–eIF4E–eIF4A complex (Fig. S3*B*). It may, however, also reflect that the excess eIF4A protein that we have in our assay to ensure saturated binding of eIF4G_682–1599_ competes for RNA binding in the competition assay. The addition of eIF4A competing for RNA binding would result in a more complicated competition analysis, which would likely explain these data. Regardless, our data establish that these fluorescently modified RNAs can be used to determine the thermodynamic framework of RNA binding to the eIF4F complex.

**Figure 1:**
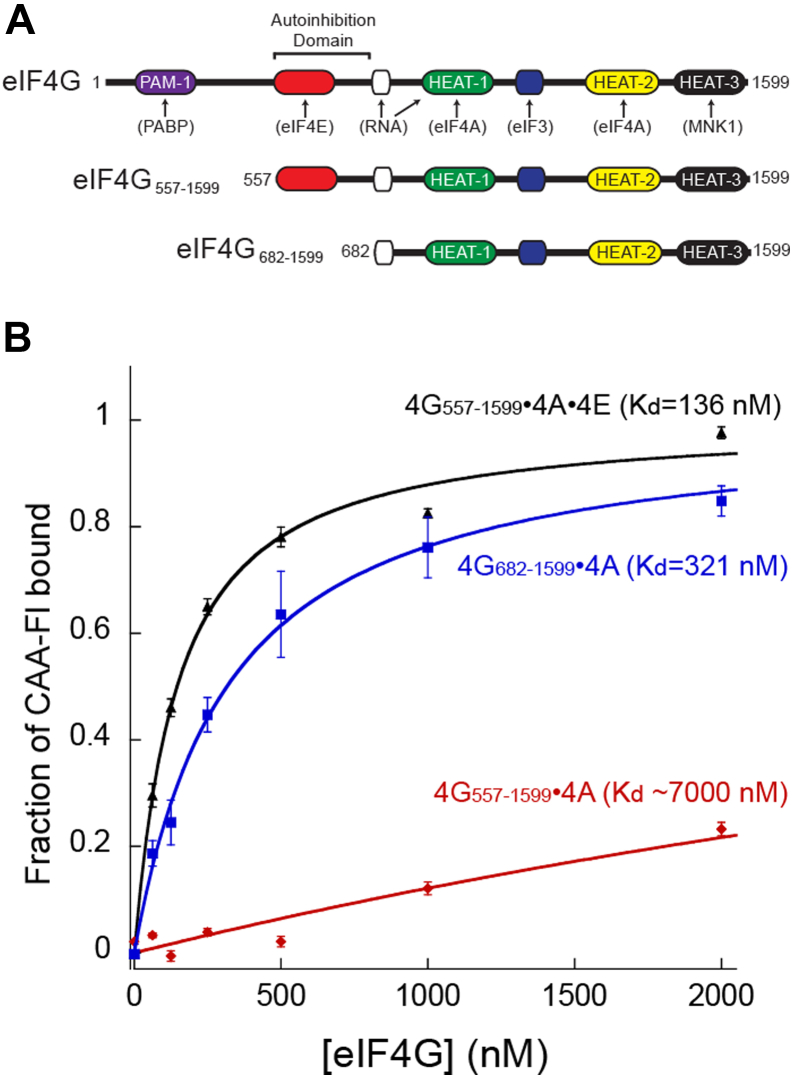
**Cap-independent role of eIF4E in RNA binding by the cap-binding complex eIF4F.***A*, *cartoon* depicting the binding domains in human eIF4G and the truncations used for this work. *B*, fluorescence polarization assay plots to determine the equilibrium dissociation constant (*K*_*d*_) of CAA42-FL to eIF4G_682–1599_ eIF4A ATP (*blue*), and eIF4G_557–1599_ eIF4A ATP in the absence (*red*), or the presence of eIF4E (*black*). The fraction of CAA42-FL bound at different concentrations of proteins is shown with each point representing the mean of three independent experiments. Error bars indicate the standard errors of the mean. eIF, eukaryotic initiation factor; FL, fluorescein labeled.

We previously identified an autoinhibitory domain located in the eIF4E-binding site domain of human eIF4G (amino acids 557–682; Fig. 1*A*) that negatively regulates the helicase activity of eIF4A in an eIF4G–eIF4A–ATP–eIF4B complex (11). To determine if this autoinhibition domain regulates cap-independent RNA binding to an eIF4G–eIF4A–ATP complex, we tested CAA-42-FL binding to eIF4G_557–1599_–eIF4A–ATP in the absence and presence of eIF4E (Fig. 1*B*). A fixed amount of CAA-42-FL RNA was incubated in the presence of a fixed amount of excess eIF4A–ATP and increasing concentrations of eIF4G_557–1599–_eIF4A–ATP complex. In the absence of eIF4E, CAA-42-FL binding to eIF4G_557–1599_–eIF4A–ATP yields a *K*_*d*_ of at least 7000 nM (Fig. 1*B* and Table 1). It is important to note that this *K*_*d*_ only reflects an estimated value because it is not possible to obtain a saturated complex with CAA-42-FL under these conditions. This is because our anisotropy assay is limited by the concentration of eIF4G_557–1599_–eIF4A–ATP that can be added without causing anomalous anisotropy signals, possibly through aggregation and nonspecific binding. In the presence of a saturating amount of eIF4E, the affinity of CAA-42-FL binding to the eIF4G_557–1599_–eIF4A–ATP is dramatically increased ∼50-fold to a *K*_*d*_ of 136 nM (Fig. 1*B* and Table 1). This shows that eIF4E converts the eIF4G_557–1599_–eIF4A–ATP into a high-affinity RNA-binding complex independent of the m^7^G cap structure.

### The autoinhibition domain in eIF4G reduces the dissociation rate constant of eIF4A from eIF4G

**Figure 2:**
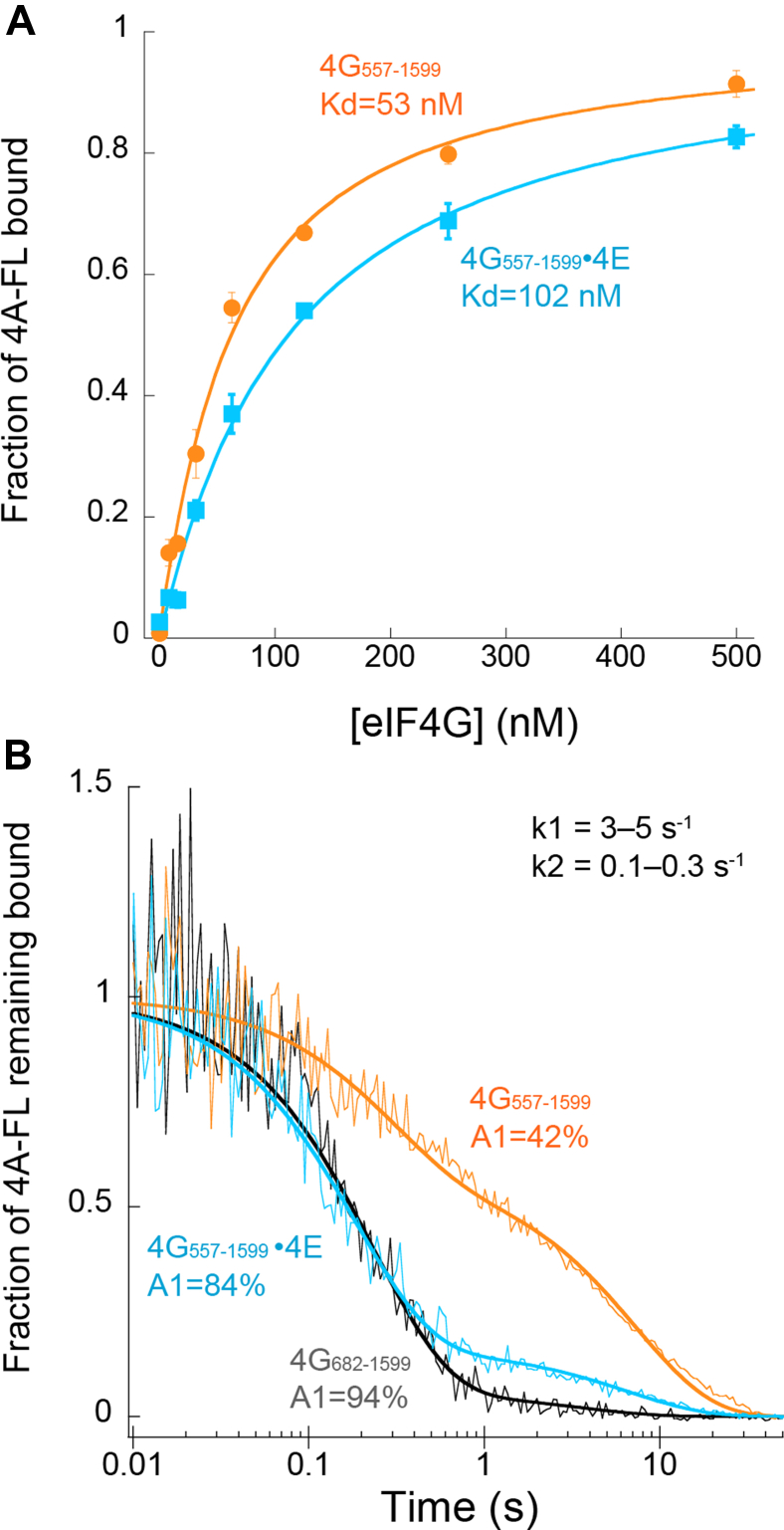
**Effect of eIF4E on affinity and dissociation kinetics of eIF4A for eIF4G.***A*, fluorescence polarization assay plots used to determine the equilibrium dissociation constant (*K*_*d*_) of eIF4A-FL for eIF4G_557–1599_ in the presence (*cyan*) or the absence (*orange*) of saturating eIF4E. The fraction of eIF4A-FL bound at different concentrations of proteins is shown with each point representing the mean of three independent experiments. Error bars indicate the standard errors of the mean. *B*, fluorescence polarization kinetic plots used to determine the dissociation rates (*k*_1_ and *k*_2_) of eIF4A-FL for eIF4G_557–1599_ in the presence (*cyan*) or the absence (*orange*) of saturating eIF4E or for eIF4G_682–1599_ (*gray*). *Thick lines* represent the fit of the data to a double exponential decay model. Data shown are the average of at least three independent experiments. eIF, eukaryotic initiation factor; FL, fluorescein labeled.

Our data show that the autoinhibition domain in human eIF4G dramatically reduces the affinity of RNA to an eIF4G–eIF4A–ATP complex (Fig. 1*B*). Moreover, our previous data showed that the autoinhibitory domain appreciably reduces the intrinsic rate of eIF4A helicase activity of the RNA-bound complex (11). To gain more insight into how the autoinhibitory domain in eIF4G can regulate eIF4A activities, we monitored the thermodynamic and kinetic parameters of eIF4A binding to eIF4G_557–1599_ in the absence and presence of eIF4E. Our recombinant eIF4G_557–1599_ possesses a stoichiometric amount of eIF4A because of the protocol that we use to exchange the insect cell eIF4A to human eIF4A (11). We therefore adapted a previously published method to use a phosphocellulose resin to remove eIF4A from the eIF4F complex (23). This method was successful in removing eIF4A from the eIF4G_557–1599_–eIF4A complex, as described in the Experimental procedures section. However, this method does result in some residual binding of eIF4E to eIF4G_557–1599_ that we estimated to be roughly 30% (Fig. S4). To obtain a fluorescently labeled eIF4A protein, we generated an FL eIF4A (eIF4A-FL) using a ligation reaction, as described in the Experimental procedures section. We first examined the binding of eIF4A-FL to an eIF4G_557–1599_–eIF4E complex by titrating eIF4G_557–1599_ into a fixed amount of eIF4A-FL and a saturating fixed concentration of eIF4E. Converting anisotropy values into the fraction of eIF4A-FL bound at each eIF4G_557–1599_ concentration yields a *K*_*d*_ of 102 nM (Fig. 2*A* and Table 1). In the absence of eIF4E, eIF4A-FL binding to eIF4G_557–1599_ yields a *K*_*d*_ of 53 nM (Fig. 2 and Table 1). Thus, eIF4E has a very modest twofold reduction in the affinity of eIF4A-FL for eIF4G_557–1599_. To gain deeper insight into the mechanism by which eIF4E may alter the way in which eIF4A interacts with eIF4G, we measured the dissociation rate of eIF4A-FL from eIF4G_557–1599_ in the absence or the presence of eIF4E. To this end, we used our anisotropy assay to carry out a stopped-flow kinetic analysis of eIF4A-FL dissociation upon the addition of a large concentration of unlabeled competitor eIF4A, as described in the Experimental procedures section. In the presence of a saturating amount of eIF4E, we observe a reduction in real-time anisotropy, which was converted to fraction of eIF4A-FL remaining bound to eIF4G_557–1599_ over time (Fig. 2*B*). The average trace of three independent experiments was found to be best fit to a double exponential decay model to calculate two dissociation rate constants (*k*-_1,4A_ and *k*-_2,4A_). The calculated intrinsic dissociation rate constants of eIF4A-FL dissociation from the eIF4G_557–1599_ complex were found to be 4 s^−1^ (*k*_1_) and 0.14 s^−1^ (k_2_). These correspond to half-lives of roughly 0.2 and 5 s, respectively, assuming first-order reactions (Fig. 2*B* and Table 2). Our data show that the faster rate constant (*k*_1_) constitutes 84% of the signal, with the remaining 16% of the signal corresponding to the slower rate constant (*k*_2_). Interestingly, when we carried out our real-time anisotropy assay using eIF4G_557–1599_ in the absence of eIF4E, we observe an appreciable change in the ratio of the signal corresponding to the two rate constants, with the faster rate constant (*k*_1_) now contributing only 42% of the signal (Fig. 2*B* and Table 2). To further calibrate these two rate constants, we also determined the dissociation rate constant of eIF4A-FL from eIF4G_682–1599_, which does not possess the autoinhibitory domain. The calculated intrinsic dissociation rate constants of eIF4A-FL dissociation from the eIF4G_682–1599_ complex were found to be essentially the same as those of eIF4A-FL dissociation from the eIF4G_557–1599_ complex (Fig. 2*B* and Table 2). The faster rate constant (*k*_1_), however, now contributes almost all the signals (94%; Fig. 2*B* and Table 2). We interpret these two intrinsic rate constants to likely reflect two different conformational states with which eIF4A can interact with eIF4G. Our data indicate that these conformational states are regulated by the autoinhibitory domain of eIF4G, with a far more stable, less dynamic, state being stabilized by the autoinhibitory domain in the absence of eIF4E.

**Table 2:** Summary of kinetic parameters

Signal	Complex bound	Nucleotide	Temperature (°C)	*k*_1_^a^ (s^−1^)	*k*_2_^a^ (s^−1^)	A_1_:A_2_^b^ (%)
CAA42-FL anisotropy	eIF4G_682–1599_ ± eIF4A	ATP	30	0.067 ± 0.001		
		—	30	>>100^c^		
	eIF4A	ATP	30	0.061 ± 0.001		
CAA42-FL fluorescence	eIF4G_682–1599_ ± eIF4A	—	25	87 ± 17		
eIF4A-FL anisotropy	eIF4G_557–1599_	—	30	3.5 ± 0.6	0.14 ± 0.01	42:58
	eIF4G_557–1599_ ± eIF4E	—	30	5.4 ± 0.6	0.15 ± 0.01	84:16
	eIF4G_682–1599_	—	30	3.3 ± 0.3	0.29 ± 0.12	94:6

a Off rates determined by real-time competition of the labeled molecule with excess amount of unlabeled species.

b Normalized amplitudes of a double-exponential dissociation.

c The value was estimated from the dead time of the stopped-flow fluorometer used.

### Contribution of the nucleotide-bound state of eIF4A on RNA binding by the eIF4F complex

**Figure 3:**
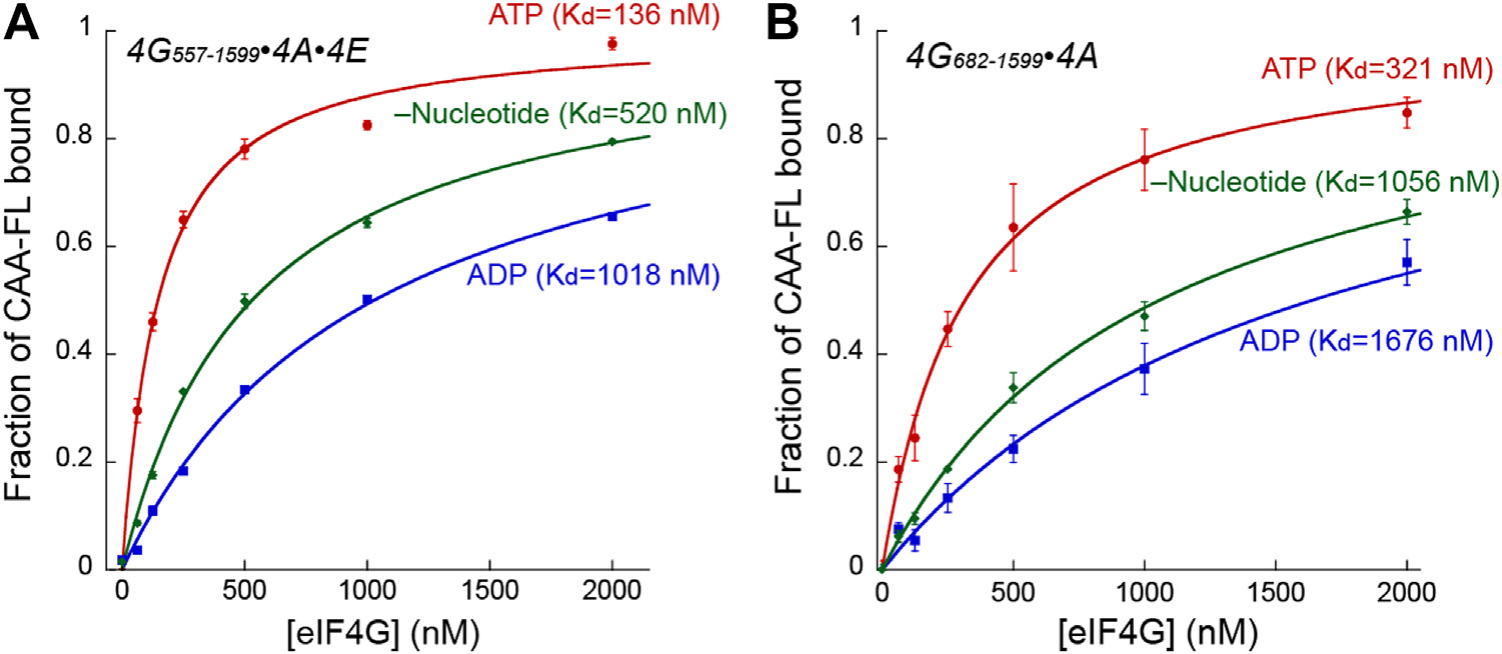
**Contribution of the nucleotide-bound state of eIF4A on RNA binding by the eIF4F complex.***A* and *B*, fluorescence polarization assay plots used to determine the equilibrium dissociation constant (*K*_*d*_) of CAA42-FL binding to eIF4G_557–1599_ eIF4A eIF4E (*A*) and eIF4G_682–1599_ eIF4A (*B*), in the absence of nucleotide (*green*), or the presence of saturating amounts of ATP (*red*) or ADP (*blue*). The fraction of CAA42-FL bound at different concentrations of proteins is shown with each point representing the mean of three independent experiments. Error bars indicate the standard errors of the mean. We highlight that to provide visual comparisons, the same curves used in Figure 1 to show eIF4G_557–1599_ eIF4A eIF4E and eIF4G_682–1599_ eIF4A in the presence of ATP (*red lines*) are reproduced in this figure. eIF, eukaryotic initiation factor; FL, fluorescein labeled.

Human eIF4A binds to RNA in an ATP-dependent manner, with a reported 40-fold higher affinity for RNA when bound to ATP *versus* ADP (16, 17). We therefore wanted to determine if the nucleotide-bound state of eIF4A also regulates RNA binding of the entire eIF4F complex. To this end, we titrated in eIF4G_557–1599_–eIF4E–eIF4A–ADP to a fixed concentration of CAA-42-FL. Compared with the data obtained in the presence of ATP (Fig. 1), we observe over a sevenfold reduction in affinity of CAA-42-FL for the eIF4F complex in the presence of ADP *versus* ATP (Fig. 3*A* and Table 1). To gain additional insight, we also titrated in eIF4G_557–1599_–eIF4E–eIF4A without nucleotide to a fixed concentration of CAA-42-FL. The affinity of CAA-42-FL to eIF4G_557–1599_–eIF4E–eIF4A in the absence of nucleotide is roughly twofold improved when compared with the presence of ADP, but the affinity is still almost fourfold reduced when compared with the presence of ATP (Fig. 3*A* and Table 1).

We decided to test whether the nucleotide-bound state of eIF4A also regulates RNA binding to the eIF4G_682–1599_–eIF4A complex. Importantly, this complex contains an eIF4G truncation that does not possess the autoinhibitory domain. Compared with the data obtained in the presence of ATP (Fig. 1), we observe over a fivefold reduction in affinity of CAA-42-FL for the eIF4G_682–1599_–eIF4A complex in the presence of ADP *versus* ATP (Fig. 3*B* and Table 1). In the absence of nucleotide, the affinity of CAA-42-FL for the eIF4G_682–1599_–eIF4A complex possesses a *K*_*d*_ of 1056 nM, which is a slight improvement in affinity compared with the complex that contains ADP (Fig. 3*B* and Table 1).

Taken together, these data indicate that the nucleotide-bound state of eIF4A regulates the interaction of RNA with the entire eIF4F complex. Similar reduction in affinity for CAA-42-FL to eIF4G_557–1599_–eIF4E–eIF4A and eIF4G_682–1599_–eIF4A is observed when ATP is replaced with ADP or no nucleotide. This indicates that the mechanism by which the nucleotide-bound state of eIF4A controls RNA-binding functions independently of the autoinhibitory domain that is controlled by eIF4E binding.

### The nucleotide-bound state of eIF4A controls the association and dissociation rate constants of RNA binding to eIF4F

**Figure 4:**
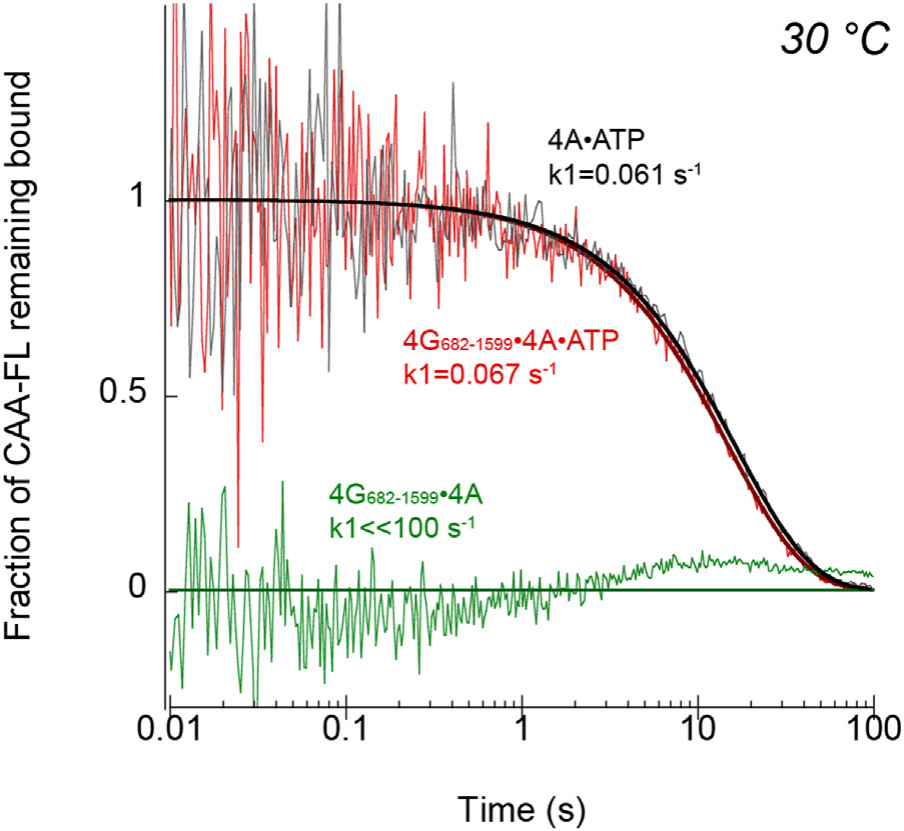
**The nucleotide-bound state of eIF4A controls the dissociation rate constant of RNA binding to eIF4F.** Fluorescence polarization kinetic plots used to determine the dissociation rate (*k*_1_) of CAA42-FL for eIF4G_682–1599_ eIF4A in the presence (*red*) or the absence (*green*) of ATP or for eIF4A in the presence of ATP (*gray*), measured at 30 °C. *Thick lines* represent the fit of the data to a single exponential decay model, except for a *green line* that represents a baseline value. Data shown are the average of at least three independent experiments. eIF, eukaryotic initiation factor; FL, fluorescein labeled.

As shown previously, we observe a roughly threefold reduction from 1056 to 321 nM in the equilibrium dissociation constant for CAA-42-FL binding to the eIF4G_682–1599_–eIF4A complex compared with CAA-42-FL binding to the eIF4G_682–1599_–eIF4A–ATP complex (Fig. 3 and Table 1). To gain a deeper understanding into what this modest change in affinity means for the way that eIF4G_682–1599_–eIF4A interacts with RNA, we measured the dissociation rate of RNA from eIF4G_682–1599_–eIF4A in the absence or the presence of ATP. To this end, we used our anisotropy assay to carry out a stopped-flow kinetic analysis of CAA-42-FL dissociation upon the addition of a large concentration of unlabeled competitor CAA-42, as described in the Experimental procedures section. In the presence of ATP, we observe a reduction in real-time anisotropy, which was converted to fraction of CAA-42-FL remaining bound to eIF4G_682–1599_–eIF4A–ATP over time (Fig. 4). The average trace of three independent experiments was found to be best fit to a single exponential decay model to calculate the dissociation rate constant (*k*_-1,RNA_). The calculated intrinsic dissociation rate constant of CAA-42-FL RNA dissociation from the eIF4G_682–1599_–eIF4A–ATP complex was found to be 0.067 s^−1^, corresponding to a half-life of roughly 10 s assuming a first-order reaction (Fig. 4 and Table 2). In contrast, when we carried out our real-time anisotropy assay using eIF4G_682–1599_–eIF4A in the absence of nucleotide, we observe what appears to be an immediate complete loss of anisotropy (Fig. 4). The observed change in anisotropy is completely dependent on the addition of the competitor RNA but is beyond the dead time (1 ms) of our real-time anisotropy assay (Fig. S5*A*). Therefore, we cannot determine the dissociation rate constant of RNA dissociation from eIF4G_682–1599_–eIF4A using these data, but we can estimate that it must be faster than 100 s^−1^ (Fig. 4). In an attempt to gain some insight into the rapid dissociation rate of CAA-42-FL from eIF4G_682–1599_–eIF4A, we also monitored the change in total fluorescence intensity over time. At a reduced temperature of 25 °C, we do observe a change in total fluorescence intensity in the presence of unlabeled competitor CAA-42 RNA (Fig. S5*B*). This change in fluorescence is indicative of CAA-42-FL dissociation and can be fit to a single exponential rate equation, as described in the Experimental procedures section. The calculated intrinsic dissociation rate constant of CAA-42-FL RNA dissociation from the eIF4G_682–1599_–eIF4A complex using this approach was found to be 87 s^−1^, which appears to be consistent with an estimated rate constant of at least 100 s^−1^ at 30 °C (Fig. 4). Our data therefore indicate that the dissociation rate of CAA-42-FL from eIF4G_682–1599_–eIF4A increases by roughly four orders of magnitude in the absence of nucleotide compared with the presence of ATP. Since the *K*_*d*_ of CAA-42-FL to eIF4G_682–1599_–eIF4A only decreases by roughly threefold in the absence of nucleotide compared with ATP, the association rate must also increase dramatically in the absence of ATP compared with the ATP-bound state (*i.e.*, the equilibrium dissociation constant is equal to the dissociation rate constant divided by the association rate constant). This suggests that in the absence of ATP, eIF4F becomes a much more dynamic complex with regard to the binding and release of RNA.

We also used our real-time anisotropy assay to determine the dissociation rate constant of CAA-42-FL from eIF4A–ATP to gain insight into the role of eIF4G in binding and releasing RNA. Interestingly, the dissociation rate constant of CAA-42-FL from eIF4A–ATP is essentially the same as that found for eIF4G_682–1599_–eIF4A–ATP (Fig. 4 and Table 2). In light of the fact that we observe *K*_*d*_ values of 4127 and 321 nM for the dissociation rate constant of eIF4A–ATP and eIF4G_682–1599_–eIF4A–ATP, respectively, this implies that eIF4G increases the association rate constant of RNA to the eIF4F complex by an order of magnitude.

## Discussion

**Figure 5:**
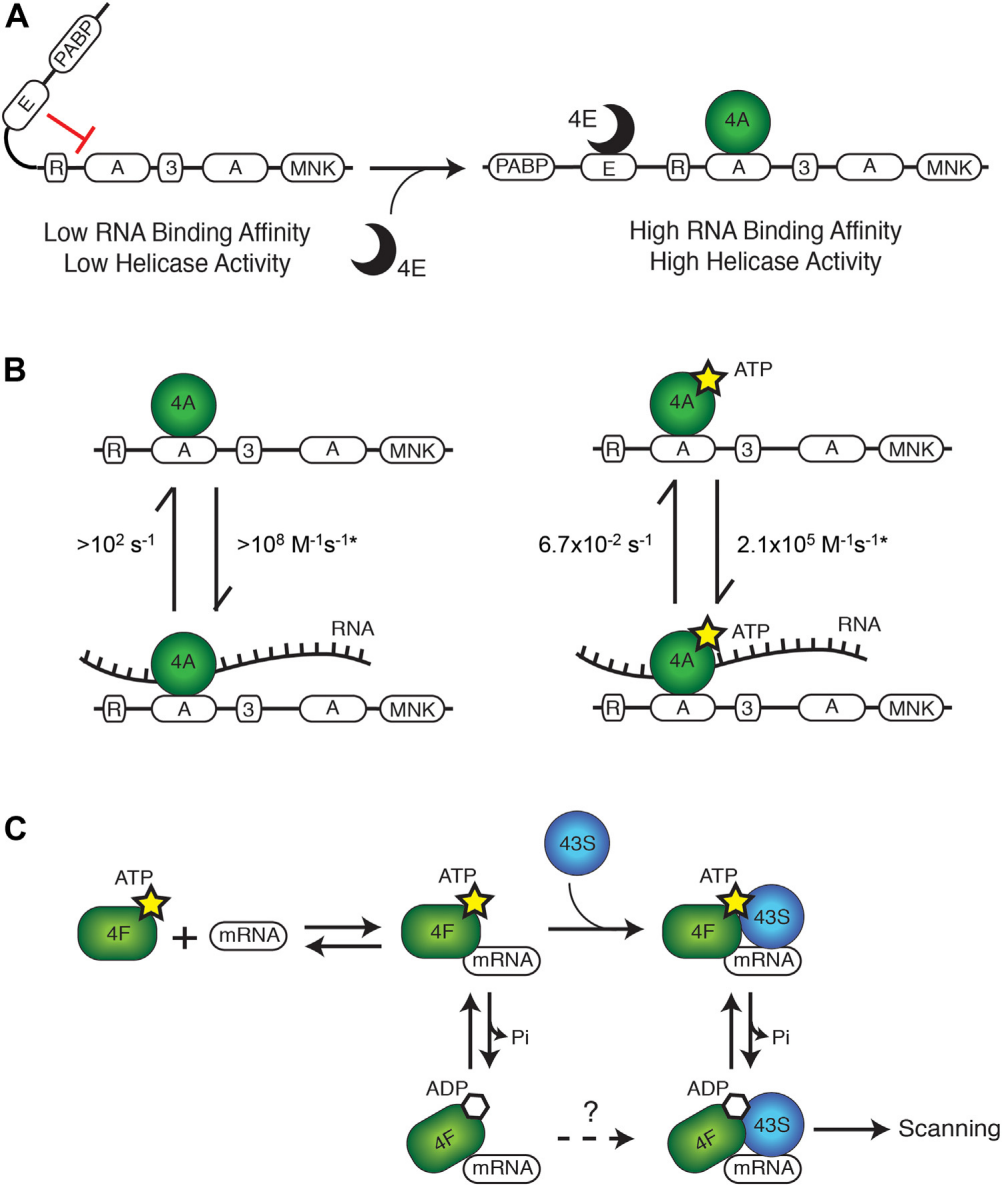
**Proposed model for RNA binding by eIF4F through a cap-independent function of eIF4E and the nucleotide-bound state of eIF4A.***A*, in the absence of eIF4E, the eIF4E-binding domain stabilizes a conformation of eIF4G that possesses low RNA-binding affinity and low eIF4A helicase activity. The binding of eIF4E stabilizes a conformation of eIF4G that possesses a high RNA-binding affinity and high eIF4A helicase activity. *B*, the hyperactive truncation of eIF4G_682–1599_ without the autoinhibitory eIF4E-binding domain is depicted in the presence of eIF4A and either no nucleotide (*left panel*) or ATP (*right panel*). In the absence of nucleotide, RNA binding and release from the eIF4G_682–1599_–eIF4A complex are both *fast*; the dissociation rate of RNA from eIF4G_682–1599_–eIF4A is estimated to be greater than ∼100 s^−1^, with a calculated association rate (∗) of greater than 1 × 10^8^ M^−1^ s^−1^ (*left panel*). In the presence of nucleotide, RNA binding and release from the eIF4G_682–1599_–eIF4A complex is *slow*; the dissociation rate of RNA from eIF4G_682–1599_–eIF4A is 0.067 s^−1^, with a calculated association rate (∗) of 2.1 × 10^5^ M^−1^ s^−1^ (*right panel*). Calculated association rates are determined using equilibrium dissociation constants (Table 1) and observed dissociation rates (Table 2). *C*, a model to depict possible intermediates during the recruitment of mRNA to the 43S PIC and its subsequent scanning. The eIF4F complex bound to ATP can form a long-lived complex with mRNA. This complex can bind to the 43S PIC to form the 48S PIC. Hydrolysis of ATP and release of phosphate converts the eIF4F complex into a complex that rapidly releases from the mRNA, which can promote the scanning/translocation of the 48S PIC. Binding of a new ATP molecule will convert eIF4F back into a long-lived RNA-binding state, which will arrest scanning of the 48S PIC until the next hydrolysis event. It is unknown whether the hydrolysis of ATP prior to 43S PIC binding enables productive 43S PIC binding (*dotted arrow* with *question mark*). eIF, eukaryotic initiation factor; PIC, preinitiation complex.

In this study, we have used steady-state and real-time anisotropy assays with a highly purified reconstituted human system to explore how interactions between eIF4E, eIF4G, eIF4A, and ATP regulate RNA binding and release from eIF4F. A summary depicting how these interactions regulate RNA binding based on our data is shown in Figure 5. Our data reveal central roles of eIF4E and the nucleotide-bound state of eIF4A in regulating the interaction of RNA with the human eIF4F complex. We focused our study on the m^7^G cap-independent interaction of RNA with eIF4F since this interaction must be regulated throughout the initiation pathway. Purification of eIF4F is complicated by the fact that it typically contains variable amounts of eIF4A and eIF4E proteins. We have circumvented this problem by using highly purified recombinant protein constructs that enable us to precisely vary each of the eIF4F components.

It has been shown that the binding of eIF4E to an autoinhibitory domain in human eIF4G strongly promotes the helicase activity of eIF4A (11, 12). Yet, this corresponded to a very little change in the apparent affinity of the eIF4F complex for an uncapped duplex substrate (11). However, the reported helicase assays were carried out in the presence of the eIF4B accessory factor, which could have masked a possible role of eIF4E in RNA-binding affinity by eIF4F. Consistent with this possibility, it has previously been shown that eIF4B dramatically increases the RNA-binding affinity of free eIF4A to RNA (24). Nevertheless, we note that eIF4B did not appear to change the RNA-binding affinity of the eIF4F complex in the same study, making it unclear whether eIF4B fundamentally changes the way that eIF4F binds to RNA. Recently, eIF4E binding to the autoinhibitory domain of plant eIF4G has been shown to increase the affinity of eIF4G for the 3′ cap-independent BTE by up to sixfold (21). Consistent with this observation, our data also demonstrate that eIF4E strongly increases the affinity of an uncapped and unstructured CAA repeat containing RNA for human eIF4F by at least 50-fold in the presence of ATP (Fig. 5*A* and Table 1). This change in affinity for RNA to human eIF4F is an order of magnitude greater than that found for plant eIF4G/4E binding to the BTE (21). The reason for this difference is not clear, but it is possible that eIF4A, which was not included in the plant-binding experiments, may be responsible. It is also interesting to note, however, that the affinity of human eIF4G/4A to domain V of the poliovirus internal ribosome entry site also increases by roughly fivefold in the presence of eIF4E (12). Thus, the magnitude of change in affinity induced by eIF4E may depend on the type of RNA that the complex interacts with. It is possible, for example, that the interaction of eIF4F with viral internal ribosome entry sites may exploit a different interaction mechanism that is less sensitive to eIF4E binding. What is the biological significance of the affinity changes observed here? Assuming the physiological concentrations of mammalian initiation factors are at least 1000 nM (25), our data indicate that the binding of uncapped RNA to human eIF4F will require eIF4E to ensure saturation.

Our data demonstrate that eIF4E binding to the autoinhibitory domain in eIF4G displays a modest twofold positive-binding cooperativity with eIF4A binding to eIF4G (Fig. 2 and Table 1). Monitoring the kinetics of eIF4A dissociation from the eIF4F complex further reveals that eIF4E increases the dissociation rate of eIF4A from eIF4G by roughly 20-fold (Fig. 2 and Table 2). Thus, eIF4E binding promotes a far more dynamic eIF4A-binding state in eIF4G. This provides a plausible mechanism with which to explain how eIF4E binding to eIF4G promotes the helicase activity of eIF4A (11), a function that requires a cycle of opening and closing of the eIF4A RecA homology domains (19, 20).

Previous studies have shown that ATP binding and hydrolysis produces a cycle of conformational and RNA-affinity changes in eIF4A (16, 17, 18). These conformational changes provide the mechanism by which eIF4A can bind and release RNA. What was not clear, however, was whether the mechanism by which eIF4A binds and releases RNA also regulates the interaction of the entire eIF4F complex, which includes other RNA-binding domains in eIF4G. The affinity of human eIF4A for RNA is 40-fold greater when the protein is bound to ATP compared with ADP (17). Our data demonstrate that the changes in affinity for RNA binding to ATP- *versus* ADP-bound states of eIF4G_557–1599_/4A/4E and eIF4G_682–1599_/4A are only sevenfold and fivefold, respectively (Fig. 3 and Table 1). We also highlight that there is an even smaller change in affinity for RNA binding to eIF4F in the absence of any nucleotide compared with the ATP-bound state (approximately threefold; Fig. 3 and Table 1). Using a filter-binding assay, no significant effect on globin or poly(A) binding to eIF4F was observed in the absence or the presence of ATP (3). The much smaller difference in affinity between nucleotide-bound states of eIF4F compared with eIF4A alone may therefore reflect the additional contribution that eIF4G has on eIF4F-binding RNA. To gain more insight into what nucleotide-bound state changes in RNA-binding affinity to eIF4F mean, we determined the dissociation rate constant to reveal how ATP hydrolysis regulates RNA binding by eIF4F. Our data reveal a striking 4-order of magnitude increase in the dissociation rate constant of RNA from eIF4F in the absence *versus* the presence of ATP (Fig. 5*B* and Table 2). Considering the corresponding approximately threefold change in affinity of RNA for eIF4F in the absence *versus* the presence of ATP, our data enable us to calculate that the association rate constant of RNA binding to eIF4F must also change by at least an order of magnitude. Specifically, the calculated association rate constant for eIF4F binding to RNA in the absence of ATP is >10^8^ M^−1^ s^−1^ *versus* 2.1 × 10^5^ M^−1^ s^−1^ in the presence of ATP (Fig. 5*B*). Thus, our data reveal that the ATP hydrolysis cycle generates a dramatic change in the rate of binding and release of RNA and eIF4F (Fig. 5*B*). The fact that the association and dissociation rates of RNA binding to eIF4F are both altered by ATP helps to explain why previous studies did not detect any ATP-dependent change in the affinity of RNA for eIF4F (3).

Based on our data and published work, we can propose a model to describe the possible complexes that can form during the recruitment of mRNA to the 40S subunit (Fig. 5*C*). The binding of ATP to eIF4F generates a high-affinity binding complex for mRNA that has a biologically long half-life (seconds) for the mRNA. Mammalian eIF4G has been shown to increase the rate of ATP hydrolysis by eIF4A roughly 10-fold (5, 26). Yet, the rate of ATP hydrolysis of eIF4F is still relatively slow, which would facilitate the association with the 43S PIC prior to ATP hydrolysis and release of mRNA from eIF4F (reviewed in Ref. (1)). Once bound to the 43S PIC to form the 48S complex, the hydrolysis of ATP and release of phosphate would generate an eIF4F complex that rapidly releases from the mRNA. This state would presumably enable scanning/translocation of the 48S complex. Binding of a new ATP molecule would cycle eIF4F back to a long-lived RNA-binding state. Whether the ADP-bound state of eIF4F is a productive complex for binding the 43S PIC is unknown (Fig. 5*C*). Interestingly, it has been reported that the rate of ATP hydrolysis by eIF4A is stimulated by the 43S PIC independently of eIF4F (27). This may imply that the functional hydrolysis of ATP occurs after the 43S PIC is recruited to the mRNA *via* the eIF4F complex. This may help to ensure that scanning/translocation of the eIF4F complex only occurs in the presence of the 43S PIC.

Our data reveal that the dissociation rate constant of RNA from eIF4A in the presence of ATP is essentially the same as that for its dissociation from eIF4G_682–1599_–eIF4A (Fig. 4 and Table 2). Yet, the affinity of RNA for eIF4A *versus* the eIF4G_682–1599_–eIF4A complex in the presence of ATP is roughly an order of magnitude less (Table 1). Thus, the association rate of RNA for eIF4G_682–1599_–eIF4A can be calculated to be ∼10-fold faster than eIF4A in the presence of ATP. Thus, eIF4G functions to appreciably accelerate the binding of RNA to eIF4F, which is likely important during mRNA recruitment to the 43S PIC and to promote scanning.

Overall, the thermodynamic and kinetic frameworks that we present here provide a more complete quantitative understanding of how eIF4F binds and releases RNA during the ATP hydrolysis cycle. It is important to note that many of our assays contain an excess of eIF4A to ensure saturation of eIF4G constructs. We can monitor eIF4G-specific changes in our experiments by subtracting any effect of free eIF4A (detailed in the Experimental procedures section). In the absence of eIF4B, an excess of eIF4A has not been observed to appreciably alter RNA binding or ATPase activity of the eIF4F complex (24). It will be important in the future to determine how the m^7^G cap structure might regulate eIF4F binding to an mRNA. In addition, it will be essential to determine how eIF4F binds and releases mRNA once it is part of the 48S complex. This is particularly important given the recently published structure of the 48S PIC (28), which indicates that eIF3 subunits are positioned in the 48S complex whereby they could interact with the eIF4A component of eIF4F during scanning. Finally, any kinetic effect that eIF4B may have on regulating eIF4F binding and release from RNA will also be important to investigate. This is critical since eIF4B is important in regulating the ATPase and helicase activities of eIF4A in the absence and presence of eIF4G (4, 5, 11, 29, 30, 31, 32, 33).

## Experimental procedures

### Sample preparations

Human eIF4A1, eIF4E, and eIF4G_682–1599_ were expressed in *Escherichia coli* BL21 (DE3), human eIF4G_557–1599_ was expressed in Sf9 insect cells as described previously (5, 11). The C-terminal fluorescent-labeled eIF4A was prepared as essentially described previously for the C-terminal-labeled eIF1 with some modifications (34). Briefly, human eIF4A1 was expressed in BL21 (DE3) as a C-terminal fusion with the C-His_6_-tagged Mxe GyrA intein. To increase efficiencies of intein cleavage and ligation, the construct had a single phenylalanine residue inserted between the C terminus of eIF4A1 and the intein. The protein was purified from the cell lysate using nickel–nitrilotriacetic acid (Ni–NTA) resin and then cleaved by incubating with 0.5 M sodium 2-mercaptoethanesulfonate (MESNA) overnight at 4 °C, yielding ∼70% cleavage. The protein was precipitated and washed twice with 3 M ammonium sulfate to remove excess MESNA and imidazole and then resuspended and passed through Ni–NTA resin again to remove the uncleaved and cleaved intein. The protein was further purified with Q Sepharose resin (GE Healthcare) in a buffer supplemented with 10 mM MESNA. The resulting eIF4A–MESNA (2.5 nmol) conjugate was ligated with 1 mM NH2-Cys-Lys(6-FAM)-COOH synthetic dipeptide (Thermo Fisher Scientific) in the presence of 0.4 M 4-mercaptophenylacetic acid, 10 mM Tris(2-carboxyethyl)phosphine, 200 mM Hepes–KOH, pH 7.0, and 200 mM KCl in 50 μl reaction. The mixture was incubated overnight at 4 °C, yielding >80% labeling efficiency. Free dipeptide was diluted through Amicon Ultra 30 kDa molecular weight cutoff (Millipore) by repeatedly adding buffer until 490 nm absorbance of the flowthrough reached below 0.1 absorbance.

To deplete the human eIF4G_557–1599_–eIF4A complex of eIF4A, we adapted a previously published protocol that uses phosphocellulose (P-11) resin (Whatman PLC) to separate eIF4A from eIF4G (35). Phosphocellulose resin was prepared by extensive washing according to the manufacturer’s guidelines (35). Human eIF4G_557–1599_–eIF4A was expressed in Sf9 cells as described previously (11). The Sf9 cell extract was passed through Ni–NTA Superflow resin and eluted into buffer A (buffer A: 20 mM Hepes, pH 7.5; 200 mM KCl; 200 mM imidazole; 10 mM 2-mercaptoethanol, and 10% glycerol). After verifying the integrity of the protein using SDS-PAGE, a stoichiometric amount of purified recombinant eIF4E (11) was added to the elution and dialyzed into buffer B (buffer B: 20 mM Hepes, pH 7.5; 100 mM KCl; 1 mM DTT, and 10% glycerol) for 2 h at 4 °C. We equilibrated 5 ml of phosphocellulose resin with 20 ml buffer B at 4 °C and applied the dialyzed eIF4G_557–1599_–eIF4A Ni–NTA elution. The column was washed twice with ice-cold 20 ml buffer B to remove eIF4A and then eluted with 15 ml buffer C (20 mM Hepes, pH 7.5; 400 mM KCl; 1 mM DTT, and 10% glycerol). A 10-fold excess of purified recombinant 4E-BP1 (11) was added to the elution and dialyzed into buffer B for 4 h at 4 °C. A heparin column (GE Healthcare) was then used to purify the protein, and fractions containing eIF4G_557–1599_ were pooled. The fractions were dialyzed into buffer B for 4 h at 4 °C with a 10-fold excess of purified recombinant 4E-BP1 added. A Mono Q column (GE Healthcare) was then used to purify the protein, and fractions containing eIF4G_557–1599_ were pooled with an additional 10-fold excess of purified 4E-BP1 added. Size-exclusion chromatography (Superose 6 16/60; GE Healthcare) was then used to purify eIF4G_557–1599_, and the fractions containing the protein were concentrated by using an Amicon Ultra unit (Millipore). The purified eIF4G_557–1599_ does not appear to have any copurifying eIF4A (as judged by SDS-PAGE), but it does copurify with some recombinant eIF4E. We estimate eIF4E to be present at roughly 30% of the stoichiometry of eIF4G_557–1599_ (Fig. S4).

RNAs were transcribed by using T7 RNA polymerase and then purified using a denaturing gel as described previously (36). The 3′ end of CAA repeat RNAs was oxidized and labeled with fluorescein as described previously (12).

### Fluorescence anisotropy–binding assay

Fluorescence anisotropy was measured in a VICTOR X5 plate reader (PerkinElmer) as described previously (34). For the equilibrium-binding assay, the reaction buffer contained 20 mM Tris acetate (pH 7.5), 70 mM KCl, 2 mM free Mg^2+^ (supplemented as MgCl_2_), 0.1 mM spermidine, 1 mM DTT, 10% glycerol, and 0.1 mg/ml bovine serum albumin, in the presence or the absence of 2 mM ATP–Mg or ADP–Mg. The final concentration of the fluorescent species was 20 nM for 3′-end labeled RNAs and 28 nM for C-terminal labeled eIF4A. The titration ranges varied depending on the affinity to be measured. Unless indicated otherwise, the final concentrations of other components were also saturating according to the available affinities and the titration range (2–10 μM eIF4A and 2 μM eIF4E). Saturation was verified by repeating each experiment with a different concentration of fixed component without observing a change in affinity of the fluorescent species. Note that our purified eIF4G_557–1599_ included a stoichiometric amount of copurified eIF4A. Thus, the amount of eIF4A represents the sum of copurified and additional eIF4A included in the reaction. The competition assay was performed similarly as the binding assay. The preformed complex including 20 nM labeled RNA, 1 or 2 μM eIF4G_682–1599_, and eIF4A in the presence or the absence of ATP–Mg was titrated with unlabeled RNA.

### Stopped-flow kinetic measurements

Stopped-flow measurements were performed on a KinTek AutoSF-120 with a dead time of 1 ms. FL RNA or eIF4A was excited at 480 nm with 20 nm (for anisotropy) or 5 nm (for fluorescence) slits with or without a polarizer. The temperature of the flow cell and loading syringes was maintained at 30 °C or 25 °C using a temperature-controlled circulating water bath. For RNA dissociation kinetics, 50 nM CAA42-FL was preincubated with 1 μM eIF4G_682–1599_ + 1 μM eIF4A + 2 mM ATP–Mg, with 4 μM eIF4G_682–1599_ + 4 μM eIF4A in the absence of nucleotide, or with 4 μM eIF4A + 2 mM ATP–Mg, at 37 °C for 10 min in buffer containing 20 mM Tris acetate, pH 7.5, 70 mM KCl, 2 mM MgCl_2_, 1 mM DTT, 0.1 mM spermidine, and 5% glycerol (v/v). For eIF4A dissociation kinetics, 70 nM eIF4A-FL was preincubated with 150 nM eIF4G_682–1599_ or eIF4G_557–1599_ (eIF4A free) in the presence or the absence of 2 μM eIF4E at 37 °C for 10 min in the same buffer. The preformed complex was loaded to AutoSF-120, further incubated for >5 min in the loading syringe, and then was mixed with an equal volume (20 μl per shot) of 10-fold molar excess (compared with eIF4G) of corresponding unlabeled competitor, which led to ≥90% dissociation at the endpoint. A time-dependent change in anisotropy or fluorescence was monitored through 531/40 nm band path filters with or without polarizers. In each experiment, a set of four to nine shots were averaged, and fitted to a single- or double-exponential equation. At least three sets of individual experiments were performed for each condition.

## Data availability

Data are presented within the article, and plasmids and other reagents are available for academic purposes upon request.

## Supporting information

This article contains supporting information.

## Conflict of interest

The authors declare that they have no conflicts of interest with the contents of this article.
