# Supplementary material for: Human eukaryotic initiation factor 4E (eIF4E) and the nucleotide-bound state of eIF4A regulate eIF4F binding to RNA
Source: J Biol Chem. 2022 Aug 11;298(10):102368. doi: 10.1016/j.jbc.2022.102368 (PMC9483636; doi:10.1016/j.jbc.2022.102368)
Supplement: Supplemental Figures S1–S5 [file mmc1.docx]

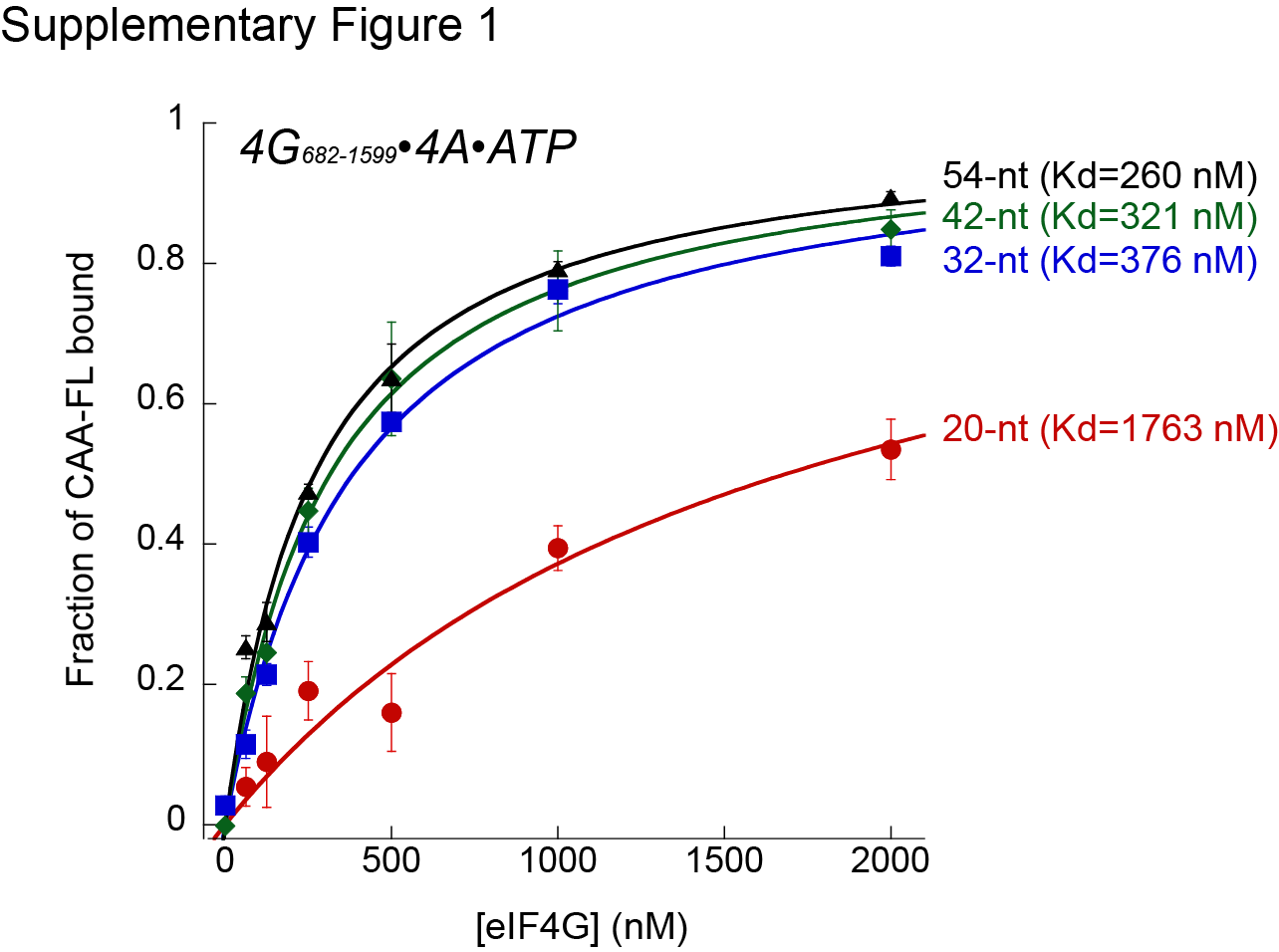


**SUPPLEMENTARY FIGURE 1. Footprint of eIF4G_682-1599_•eIF4A.** Fluorescence polarization assay plots used to determine the equilibrium dissociation constant (K_d_) of CAA-FL for eIF4G_682-1599_•eIF4A•ATP: 20 nucleotides (red), 32 nucleotides (blue), 42 nucleotides (green), and 54 nucleotides (black). The points shown in the curves are the average of at least 3 trials ± standard error of the mean.

**
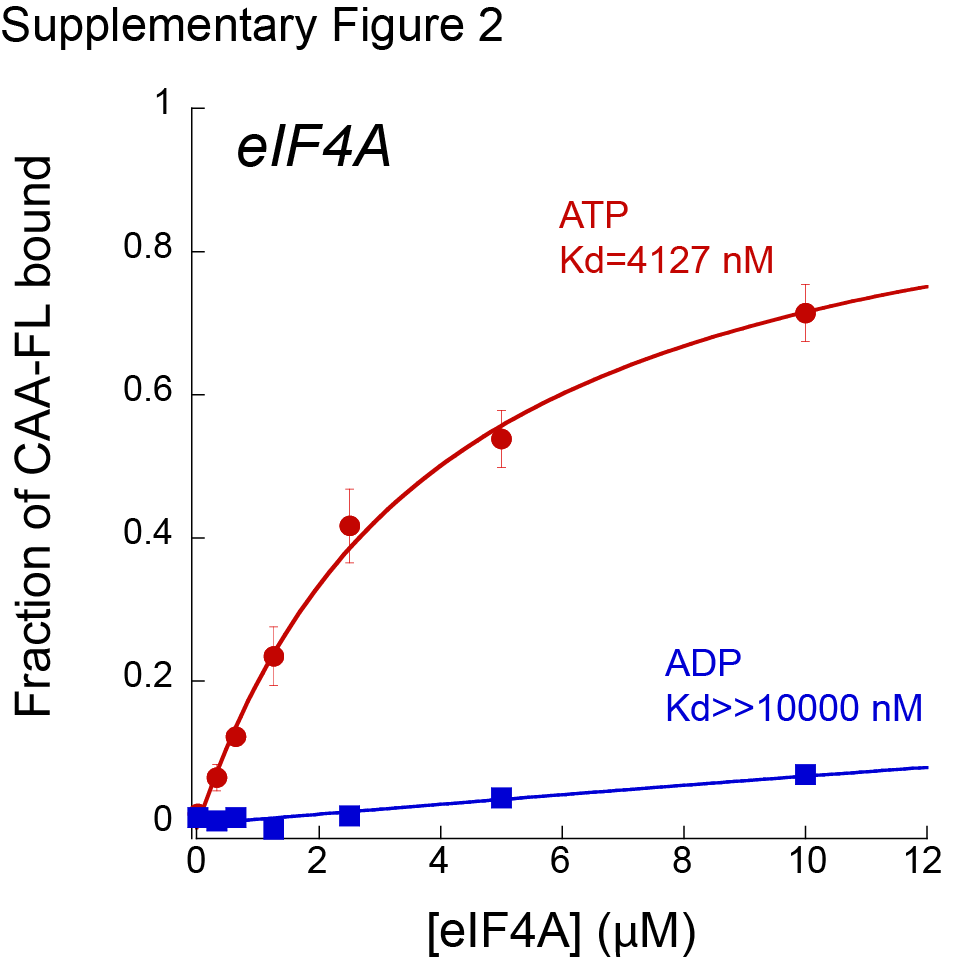
**

**SUPPLEMENTARY FIGURE 2. Affinities of CAA42-FL for eIF4A.** Fluorescence polarization assay plots used to determine the equilibrium dissociation constant (K_d_) of CAA42-FL for eIF4A in the presence of ATP (red) or ADP (blue). The points shown in the curves are the average of at least 3 trials ± standard error of the mean.


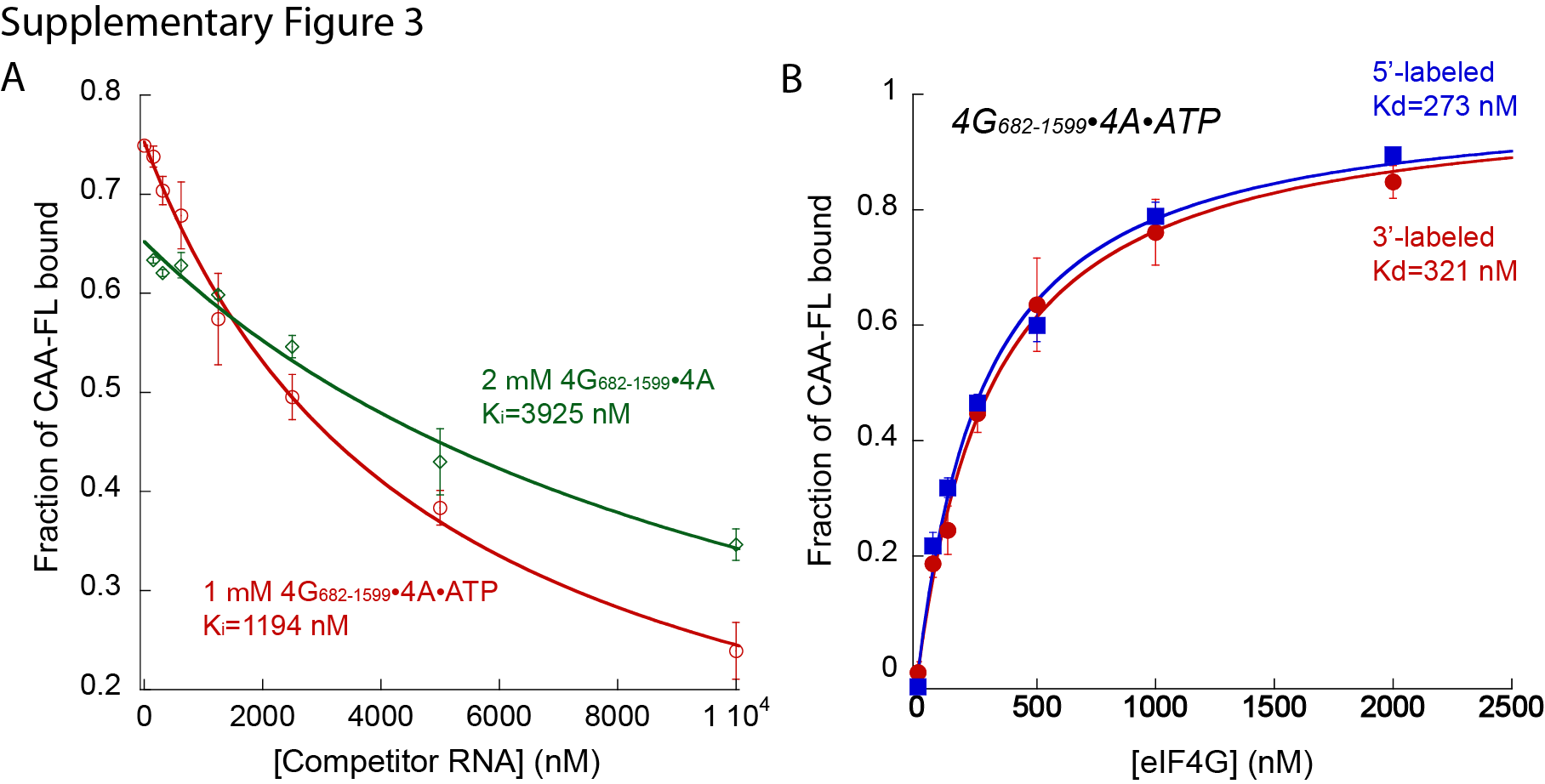


**SUPPLEMENTARY FIGURE 3. RNA competition assay and binding assay with 5′-end labeled CAA42.** A. RNA competition assay plots to determine the equilibrium inhibition constant (K_i_) of CAA42-FL for eIF4G_682-1599_•eIF4A in the presence (red) or absence (green) of ATP. B. Fluorescence polarization assay plots used to determine the equilibrium dissociation constant (K_d_) for eIF4G_682-1599_•eIF4A•ATP using CAA42 RNA labeled at 5’-end (blue) or 3’ end (red). The points shown in the curves are the average of at least 3 trials ± standard error of the mean.

Supplementary Figure 4

**
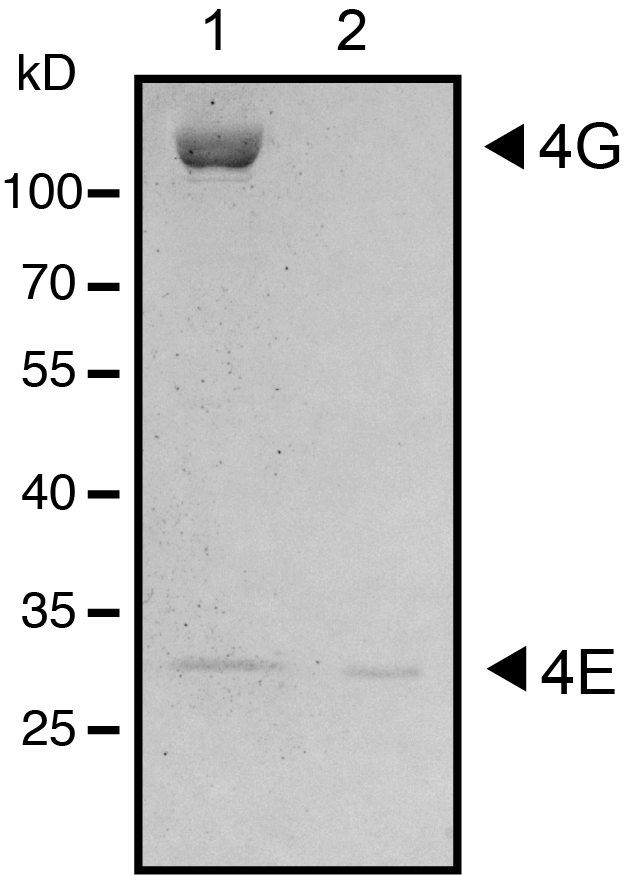
**

**SUPPLEMENTARY FIGURE 4. Purified eIF4G_557-1599_ minus eIF4A.** Coomassie stained SDS gel showing purified eIF4G_557-1599_ following the phosphocellulose purification protocol described in Experimental Procedures (lane 1). Purified human recombinant eIF4E is shown in lane 2. Relative positions of molecular weight markers are indicated on the left side of the gel and the positions of eIF4G_557-1599_ and eIF4E are indicated.

**
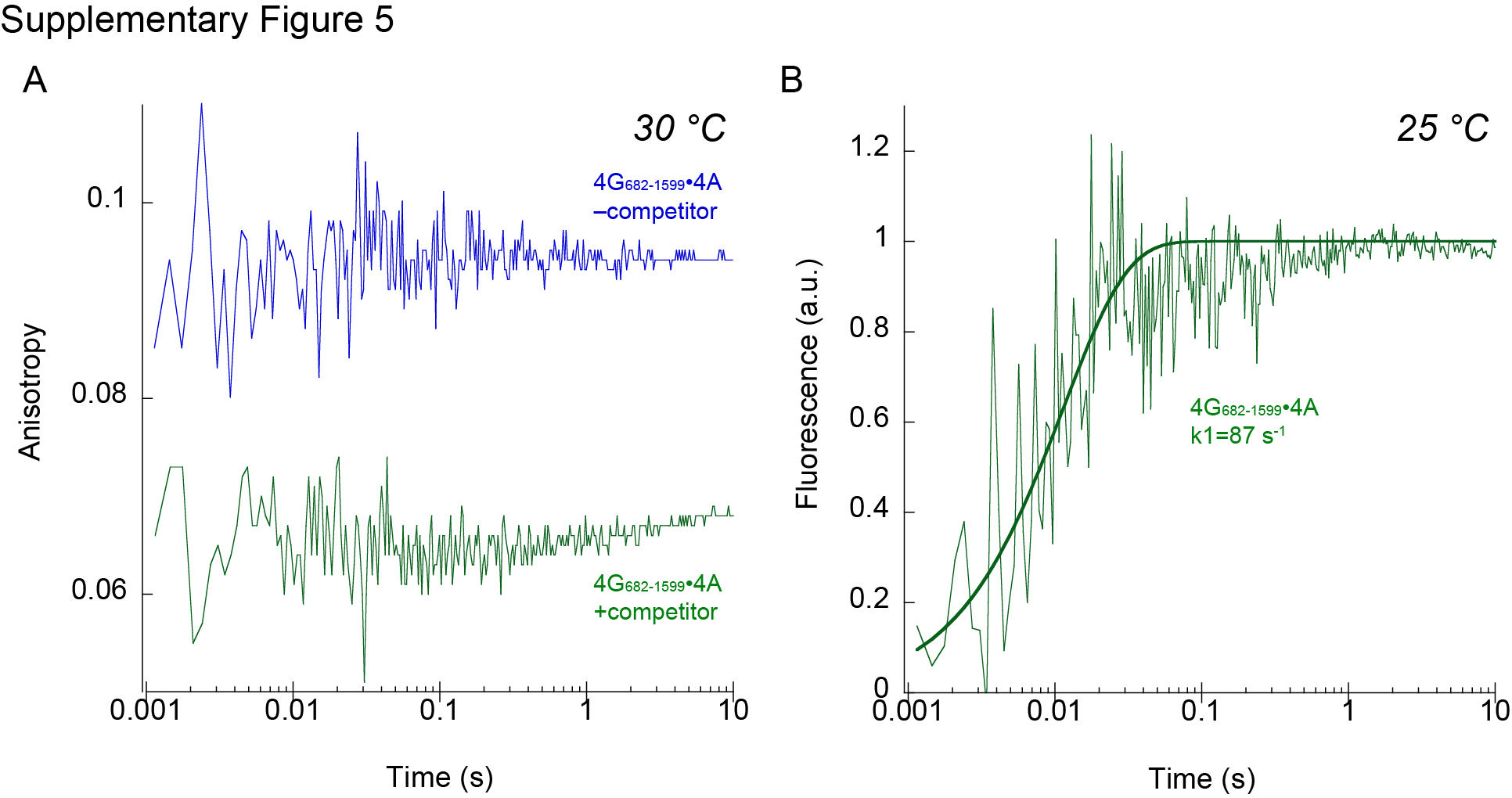
**

**SUPPLEMENTARY FIGURE 5. Dissociation kinetics of CAA42-FL for eIF4G_682-1599_•eIF4A using fluorescence intensity.** A. Fluorescence anisotropy kinetic plots of CAA42-FL for eIF4G_682-1599_•eIF4A in the absence of ATP, measured with (green) or without (blue) an addition of the competitor RNA at 30 °C. B. A fluorescence intensity kinetic plot used to determine the dissociation rate (k_1_) of CAA42-FL for eIF4G_682-1599_•eIF4A in the absence of ATP, measured at 25 °C. Thick lines represent the fit of the data to a single exponential model. Data shown is the average of at least 3 independent experiments.
